# Androgens Contribute to Sex Differences in Myocardial Remodeling under Pressure Overload by a Mechanism Involving TGF-β

**DOI:** 10.1371/journal.pone.0035635

**Published:** 2012-04-25

**Authors:** Cecilia Montalvo, Ana V. Villar, David Merino, Raquel García, Miguel Ares, Miguel Llano, Manuel Cobo, María A. Hurlé, J. Francisco Nistal

**Affiliations:** 1 Departamento de Fisiología y Farmacología, Facultad de Medicina, Universidad de Cantabria, Santander, Spain; 2 Servicio de Cirugía Cardiovascular, Hospital Universitario Marqués de Valdecilla, Santander, Spain; 3 Hospital Comarcal de Laredo, Cantabria, Spain; 4 Servicio de Cardiología, Hospital Universitario Marqués de Valdecilla, Santander, Spain; 5 Instituto de Formación e Investigación Marqués de Valdecilla (IFIMAV), Santander, Spain; Virginia Commonwealth University, United States of America

## Abstract

**Background:**

In clinical studies, myocardial remodeling in aortic valve stenosis appears to be more favorable in women than in men, even after menopause. In the present study, we assessed whether circulating androgens contribute to a less favorable myocardial remodeling under pressure overload in males. We examined sex-related differences in one-year-old male and female mice. Whereas male mice at this age exhibited circulating androgen levels within the normal range for young adults, the circulating estrogens in females were reduced. The contribution of gonadal androgens to cardiac remodeling was analyzed in a group of same-age castrated mice.

**Methodology/Principal Findings:**

Animals were subjected to transverse aortic constriction (TAC). Echocardiography was performed 2 weeks after TAC and myocardial mRNA levels of TGF-βs, Smads 2 and 3, collagens, fibronectin, β-myosin heavy chain and α-myosin heavy chain were determined by q-PCR. Protein detection of p-SMAD2/3 was performed by Western Blot. Histological staining of fibrosis was performed with picrosirius red and Masson's trichrome. Compared with females, males developed more severe tissue fibrosis, LV dilation and hemodynamic dysfunction. TAC-males showed higher myocardial expression levels of TGF-βs and the treatment with a neutralizing antibody to TGF-β prevented myocardial fibrosis development. Orchiectomy diminished TAC-induced up-regulation of TGF-βs and TGF-β target genes, and it also reduced fibrosis and hemodynamic dysfunction. The capability of androgens to induce TGF-β expression was confirmed in NIH-3T3 fibroblasts and H9C2 cardiomyocytes exposed to dihydrotestosterone.

**Conclusions/Significance:**

Our results indicate that circulating androgens are responsible for the detrimental effects in the myocardium of older male mice subjected to pressure overload through a mechanism involving TGF-βs.

## Introduction

Degenerative aortic valve stenosis (AS) is a common cardiovascular disorder and the most prevalent acquired valvular disease in Western countries [1]. In this pathology, the chronic pressure-overload condition causes left ventricular (LV) remodeling that is characterized by the hypertrophic growth of cardiomyocytes, proliferation of cardiac fibroblasts, increased deposition of extracellular matrix constituents, and loss of myocytes with fibrotic replacement. These phenomena result in LV diastolic and systolic dysfunctions and, over time, heart failure [2]. Transforming growth factors-β (TGF-β) are considered to be crucial factors in LV remodeling [3], both in animal models of pressure overload [4]–[6] and in patients with AS [7]–[10], through their regulation of the transcription of genes encoding components of the extracellular matrix in fibroblasts and sarcomeric elements in cardiomyocytes.

Sex has a profound impact on the cardiac remodeling response to pressure overload induced by AS or hypertension [9]–[15]. Hypertrophy is more frequently associated with LV dilation and systolic dysfunction in men, whereas it exhibits a more favorable geometry to preserve systolic pump performance in women. The mechanisms underlying such differences are poorly understood. Under the assumption that estrogens exert protective cardiovascular effects, a great deal of effort has been devoted to analyzing their contribution to sex-related differences in myocardial remodeling under pressure stress in young rodents [14]–[16]. Clinical studies on AS patients, however, showed that LV remodeling also occurs differently in postmenopausal women, who lack the putative estrogen-dependent cardiovascular protection, than in older men, many of whom have circulating testosterone levels that would fall within the normal range for young men [9]–[13]. This observation suggests that circulating androgens may be involved in the less favorable remodeling reported in male AS patients.

Increasing evidence suggests that androgens can exert detrimental effects on the cardiovascular system. In young humans, the widespread long-term abuse of anabolic androgenic steroids (including testosterone and its synthetic derivatives) for non-medical purposes is responsible for an alarming number of cases of a syndrome characterized by LV hypertrophy and early abnormalities of systolic and diastolic longitudinal myocardial function, which could be the consequence of myocardial cell injury and fibrotic repair [17]–[20]. Similarly, animal models show that exercise training associated with supraphysiological levels of anabolic steroids induces maladaptive remodeling and a deterioration of cardiac performance [21]. Exogenous testosterone also exerts deleterious effects on myocardial remodeling following myocardial infarction in rats [22]. A few recent reports suggest that physiological gonadal androgens play a critical role in the pathological cardiac phenotypes developed by males of several strains of genetically modified rodents [23]–[25]. Whether the detrimental effect of androgens extends to the myocardial remodeling under pressure overload remains unknown.

In this study, we postulated that gonadal-released androgens enhance the susceptibility of the heart to pressure overload, contributing to less favorable cardiac remodeling in male mice compared with females. To more closely mimic the AS clinical scenario, we used mice of an elder age range in which males exhibit circulating androgen levels within the normal range for young adults, while females display reduced circulating estrogens. Sex-related differences in LV morphology, geometry and hemodynamic performance, as well as the underlying patterns of gene expression were analyzed following transverse aortic constriction in old females and old, castrated or not, males.

## Methods

### Ethics Statement

The study was approved by the University of Cantabria Institutional Laboratory Animal Care and Use Committee (approval ID 2008/05) and conducted in strict accordance with the “European Directive for the Protection of Vertebrate Animals Used for Experimental and Other Scientific Purposes” (European Union Directive #86/606/EEC). All manipulations were performed under anesthesia and all efforts were made to minimize animal suffering.

### Animals and experimental groups

The experiments were performed in 12-month-old female and male mice (C57BL/6; n = 97) housed in a room kept at 22°C with a 12∶12 h light/dark cycle and provided with food and water *ad libitum*.

### Plasma levels of sex hormones

Plasma levels of testosterone and estradiol were measured by immunoassay with the ADVIA Centaur Immunoassay Systems (Siemens Healthcare Diagnostics Inc, USA) and GRG Estradiol ELISA kit (Germany), respectively.

### Gonadectomy

Bilateral orchiectomy was conducted in 10-month-old mice anesthetized with ketamine (10 mg/kg). To avoid any residual effects of androgens, two months were allowed between gonadectomy and TAC or sham aortic surgery.

### Transverse aortic constriction

LV pressure overload was induced by calibrated banding of the aorta at the transverse arch level (TAC), which is a well-validated experimental model [26]. Briefly, mice were anesthetized by intraperitoneal injection of ketamine (10 mg/kg) and xylazine (15 mg/kg). Surgery was performed under spontaneous ventilation. The aorta was approached extrapleurally and constricted at the mid-transverse arch level with a 7/0 polypropylene ligature using a blunted 27-gauge (0.41-mm OD) needle as a calibrator [27]. This constriction induced a similar degree of geometric stenosis in all mice (approximately 65% in diameter) since no significant sex differences in the diameter of the ascending aorta were observed in the baseline echocardiographic study (data not shown).

Mice were sham-operated or subjected to TAC for 2 weeks. After completion of the follow-up, mice were euthanized, and the cardiac mass was measured gravimetrically and indexed to the animal's body weight. LV samples were snap frozen in liquid nitrogen for RNA extraction or fixed in 4% paraformaldehyde for histology.

A series of TAC-male mice were treated during the follow-up period with a pan-neutralizing monoclonal antibody anti-TGF-β (1D11.16.8 clone) specific for all isoforms of murine TGF-β (n = 4) or with an irrelevant murine isotype-matched control immunoglobulin (IgG1) (n = 4). Mice received i.p. injections of the Ab or IgG1 at the dose of 0.5 mg every other day over the 15 day follow-up.

### Echocardiography

Transthoracic echocardiography was performed and analyzed in a blinded manner using a Vevo-770 ultrasonic system (VisualSonics, Toronto, ON, Canada) equipped with a high-resolution transducer centered at 30 MHz and specific analytic software. The studies were performed with the mice sedated with isoflurane (induction at 3% and maintenance at 0.5–2% in O_2_) and placed on a heated (37°C) platform. Body temperature, ECG and respiration were monitored. The continuous delivery of isoflurane was titrated to maintain a heart rate over 400 bpm. Figure S1 shows representative echocardiographic recordings. Transcoarctational gradients were interrogated using 2D-guided pulsed Doppler at the aortic arch immediately distal to the TAC. LV dimensions and wall thicknesses were measured following the recommendations of the American Society of Echocardiography. The mitral annular plane systolic excursion (MAPSE) was obtained from four-chamber M-mode images at the septal mitral annulus. Diastolic parameters were obtained by analysis of mitral inflow and tissue Doppler imaging. The ratio of peak early transmitral flow velocity (E) to peak early myocardial tissue velocity (E′) was used as an index of LV filling pressure. The following parameters were derived from the recordings: heart rate, pressure gradient across the arch constriction, aortic diameter at the sinotubular junction, LV end-diastolic (LVEDd), and end-systolic (LVESd) internal dimensions, interventricular septum (IVST) and posterior wall (PWT) thicknesses, and LV mass (LVM). The relative LV diastolic radius (LVEDr/PWT) was used to assess the concentricity of LV hypertrophy. The LV ejection fraction (LVEF) and mitral annular plane systolic excursion (MAPSE) were used as indices of radial and longitudinal systolic functions, respectively.

### Quantitative PCR assays

Total RNA from the LV myocardium was obtained by TRIzol extraction (Invitrogen), and 1 µg of the isolated RNA was reverse-transcribed into cDNA with a RT-PCR kit (Fermentas), according to the manufacturer's instructions. Quantitative real-time PCR was performed in a MX-3005P Stratagene thermocycler. The following TaqMan specific assays (Applied Biosystems) were used: TGF-β1, TGF-β2, TGF-β3, Smad 2, Smad 3, collagen I α1, collagen III α1, fibronectin-1 (FN), β-myosin heavy chain (β-MHC) and α-myosin heavy chain (α-MHC). Gene expression was normalized to that of the housekeeping gene ribosomal 18S rRNA, which was measured in parallel in each sample. Duplicate transcript levels were determined in a minimum of three independent experiments.

### Western Blot

To obtain myocardial nuclear fractions, LV samples were homogenized in ice-cold lysis buffer (10 mM Hepes, 100 mM KCl, 1.5 mM MgCl, pH = 7.4) containing a protease inhibitor cocktail (Sigma-Aldrich p8340). Lysates were centrifugated (4 min, 3,500 rpm, 4°C) and the resulting pellet was resuspended in nuclear buffer (10 mMHepes, 0.45 M NaCl and 1 mM EDTA, pH = 7.9), incubated 30 min in ice and centrifugated again (12 min, 13,000 rpm, 4°C). The supernatants containing the nuclear fraction were harvested. Thirty µg of nuclear protein extracts were resolved on 10% sodium dodecyl sulfate-polyacrylamide gel and transferred to polyvinylidene difluoride membranes. The membranes were incubated with primary antibodies (Santa Cruz Biotechnology) to phospho-Smad2/3 and to ELK1 (nuclear loading control). The membranes were incubated with peroxidase conjugated secondary antibodies. Immunoreactivity was detected with Advanced ECL detection reagents (GE Healthcare).

### Collagen content by light microscopy

Hearts were fixed in paraformaldehyde, cryopreserved with sucrose and frozen at −80°C while embedded in OCT compound for cryosectioning. Coronal sections (10 µm) at the level of the papillary muscles were stained with picrosirius red and viewed with polarized light under dark-field optics to detect birefringence of mature collagen fibers. Each section was photographed. Five fields were randomly selected from each of three sections per animal. Within each field, the proportion of labeled area was calculated by densitometric analysis with the ImageJ software.

### Histological assessment of myocardial fibrosis

Hearts were fixed in paraformaldehyde (4% in PBS, freshly prepared) for 48 h and, then, embedded in paraffin. The degree of fibrosis was determined in 6 µm sections stained with Masson's trichrome.

### Experiments with cell lines

NIH-3T3 fibroblasts (ATCC) and H9C2 cardiomyocytes (ATCC) were cultivated in Dulbecco's modified Eagle's medium supplemented with 10% heat-inactivated fetal bovine serum, 100 units/ml penicillin and 100 mg/ml streptomycin at 37°C in 5% CO_2_. Cells were incubated with the nonaromatizable androgenic steroid dihydrotestosterone (DHT) (8 and 15 nM) for 16 h. Total mRNA was obtained and retrotranscribed to perform qPCR for TGF-βs as described above. All assays were performed in duplicate and repeated on three separate occasions.

### Statistics

The GraphPad Prism 5.01 and PASW Statistics 18 (SPSS Inc, Chicago, IL) packages were used. Values are reported as means ± S.E.M. Student's *t*-test was used to assess differences between means of continuous variables. The influences of sex or castration and pressure overload on myocardial gene expression and echocardiographic parameters were assessed by ANOVA and repeated-measures two-way ANOVA, respectively. The Bonferroni post hoc test was used when appropriate. Regression analysis was used to detect correlations between gene expression levels. *p*<0.05 was considered significant.

## Results

### In one year mice, as compared with same sex young animals, the circulating levels of androgens in males were similar while estrogens in females were lower

The hormonal status of the one-year old mice used for this study was determined and compared with that of a reference group of 4-month-old males (n = 6) or females (n = 6). One-year-old males displayed baseline testosterone levels that were similar to those of the reference group of 4-month-old males (4.3±3.6 ng/ml vs 5.8±2.6 ng/ml; p NS). In contrast, the 17β-estradiol levels in intact one-year-old females were lower than those of 4-month-old females (7.1±2.0 pg/ml vs 37.8±13.2 pg/ml; p<0.01). In CAST-males, the testosterone levels two months after castration were below detection limits.

### Two weeks after TAC, LV geometrical and functional deterioration was more severe in intact males than in females

**Figure 1:**
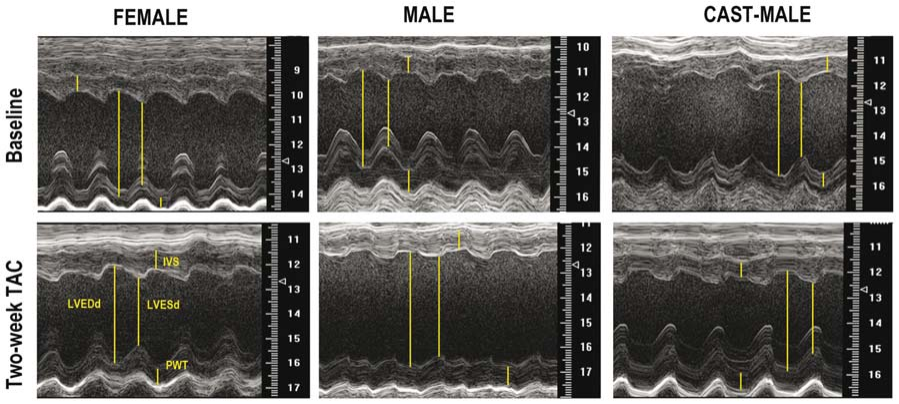
Parasternal short-axis M-mode tracings of the left ventricle obtained from representative mice of each experimental group, before (baseline) and two weeks after aortic constriction (TAC). LVESd: Left ventricular end-systolic dimension; LVEDd: Left ventricular end-diastolic dimension; PWT: posterior wall thickness; IVST: interventricular septum thickness.

Representative echocardiographic recordings are depicted in Figure 1. The baseline and post-TAC values of the echocardiographic parameters are shown in Table 1. No significant differences between groups were shown in the heart rate. Aortic constriction resulted in similar transcoarctation gradients in females and males, castrated or not (Table 1).

**Table 1:** Values of the echocardiographic parameters at baseline and two week post-TAC.

	Male				Female	
	Baseline	TAC	CAST	CAST+TAC	Baseline	TAC
**Gradient** (mmHg)	4.6±1.2	48.0±8.2+++	3.5±2.9	47.1±3.9+++	2.5±1.5	56.5±4.5+++
**Frequency** (b.p.m.)	435±12	435±25	444±14	444±20	442±14	437±19
**IVST** (mm)	0.68±0.05	0.89±0.03++	0.66±0.03	0.78±0.05	0.62±0.02	0.84±0.08++
**PWT** (mm)	0.67±0.03	0.84±0.05++	0.68±0.03	0.78±0.06	0.66±0.03	0.89±0.07++
**LVEDd** (mm)	4.0±0.1	4.3±0.2	3.6±0.1$	3.8±0.2$	3.8±0.1	3.8±0.1**
**LVESd** (mm)	2.7±0.06	3.4±0.3+++	2.1±0.1$$	2.6±0.2**^+$$^**	2.5±0.1	2.4±0.2***
**LVM-Echo** (mg)	74.5±4.4	123.2±15.9+++	59.3±3.6	84.6±9.6**^+$^**	64.0±3.1	95.4±8.1+
**LVM-Grav** (mg)	91.2±4.7^a^	115.0±4.9++	65.3±3.2^a^	84.7±5.8**^+$$^**	70.6±3.2^a^	90.5±4.2**^++^** **
**LVMI-Grav** (mg/g)	3.4±0.20^a^	4.3±0.1+++	2.8±0.21^a^	3.7±0.2**^++$$^**	3.0±0.1^a^	3.8±0.3++
**BW** (mg)	27.1±0.9	26.7±0.6	23.7±1.3	25.9±0.6	23.5±1.0	23.8±1.0

IVST: interventricular septum thickness; LVPWT: left ventricular posterior wall thickness; LVEDd: left ventricular end diastolic dimension; LVESd: left ventricular end systolic dimension; LVM-Echo: left ventricular mass by echocardiography; LVM-Grav: left ventricular mass by gravimetry; LVMI-Grav: gravimetric left ventricular mass indexed to the body weight; BW: Body weight. Values are mean ± SEM. a: Values from independent groups of mice killed at baseline.

+ p<0.05,

++ p<0.01,

+++ p<0.001, TAC vs baseline;

* p<0.05,

** p<0.01,

*** p<0.001, male vs female;

$ p<0.05,

$$ p<0.01, castrated vs intact (Bonferroni post hoc test).

**Figure 2:**
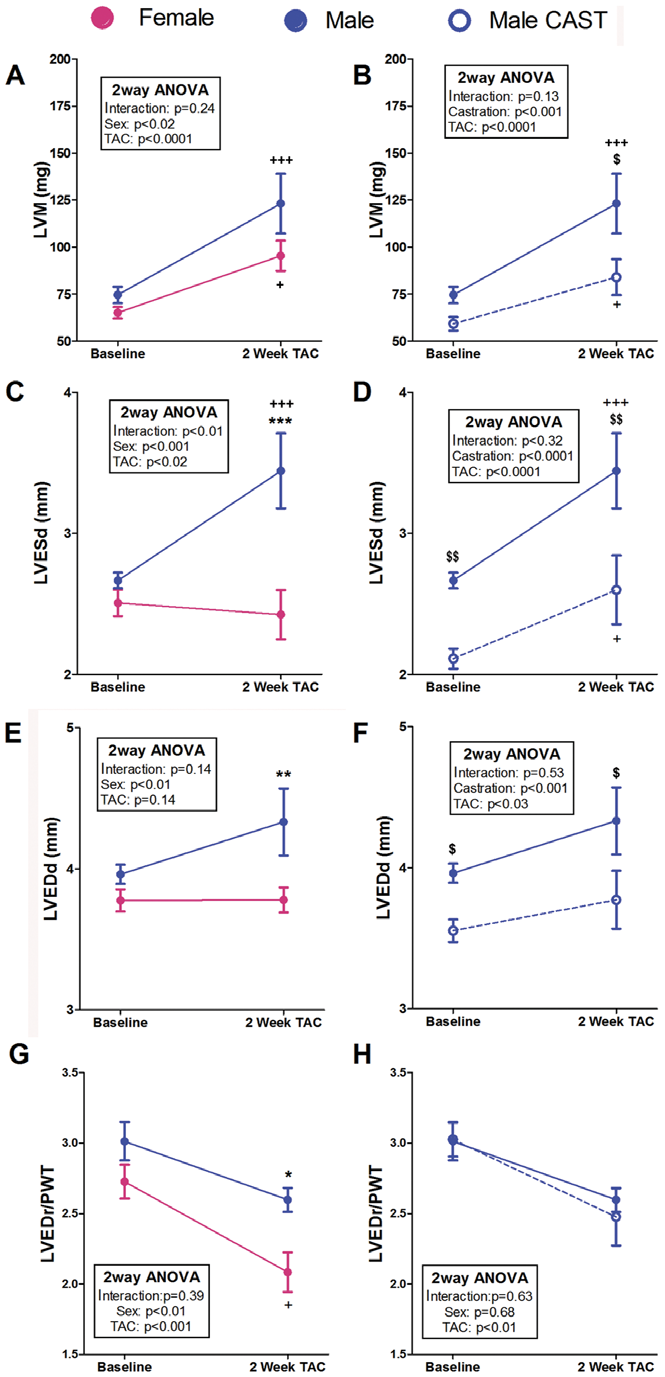
Evolution of the echocardiographic dimensional changes induced by pressure overload in mice subjected to TAC during a 2-week follow-up period. LVM: Left ventricular mass; LVESd: Left ventricular end-systolic dimension; LVEDd: Left ventricular end-diastolic dimension. LVEDr/PWT: Relative left ventricular end-diastolic radius. Data are expressed as mean ± SEM. Males vs. females: **p<0.01, ***p<0.001; TAC vs. same group baseline: ^++^p<0.01, ^+++^p<0.001; CAST vs. intact: $p<0.05, $$p<0.01. (Repeated-measures two-way ANOVA and Bonferroni post-hoc test).

Two weeks after TAC, sex-related differences in LV geometry and function were apparent in the intact mice. Females did not show any significant change of LVED and LVES dimensions after TAC (Figs. 1 and 2). Conversely, males displayed a trend towards dilation of both LV dimensions and reached significantly higher LVED and LVES dimensions than females two weeks after TAC (Figs. 1 and 2). The PWTs at baseline and their increases after TAC were similar in both sexes (Table 1) and the LVEDr/PWT after surgery was lower in TAC-females than in TAC-males (Fig. 2), indicating a significantly higher concentricity in females as a consequence of their lack of LV dilation.

**Figure 3:**
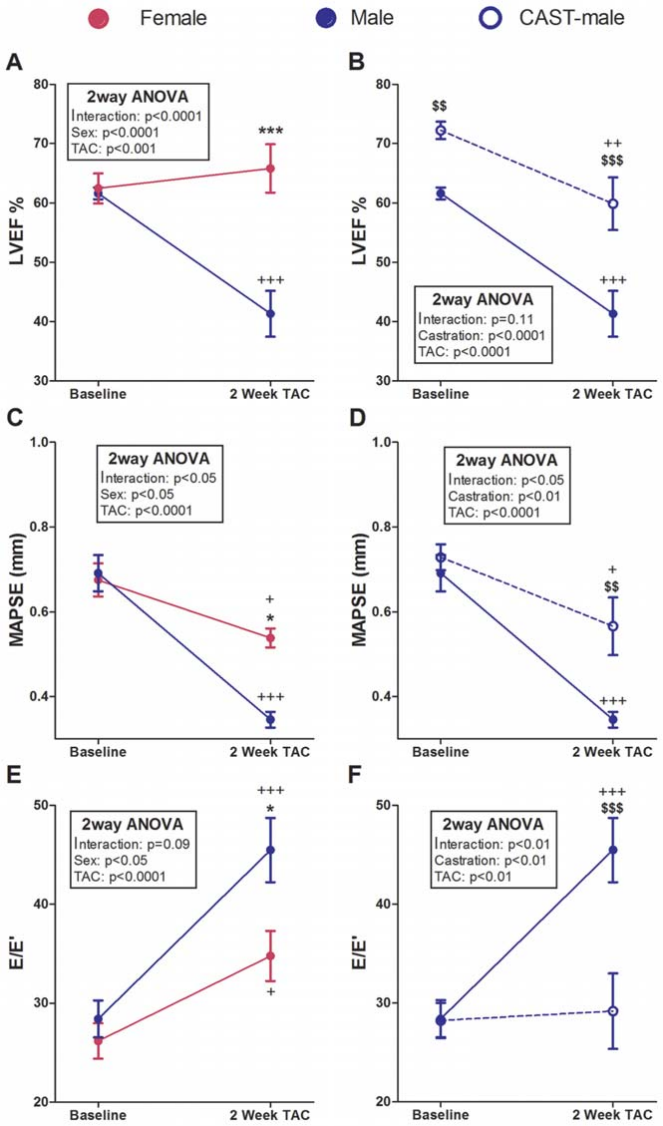
Evolution of the echocardiographic functional changes induced by pressure overload during a 2-week follow-up period in mice subjected to TAC. LVEF: Left ventricular ejection fraction; MAPSE: Mitral annular plane systolic excursion. E/E′: Peak early transmitral flow velocity (E) to peak early myocardial tissue velocity (E′) ratio. Data are expressed as mean ± SEM. Males vs. females: *p<0.05, ***p<0.001; TAC vs. same group baseline: ^+^p<0.05, ^+++^p<0.001; CAST vs. intact: $$p<0.01, $$$p<0.001. (Repeated-measures two-way ANOVA and Bonferroni post-hoc test).

Systolic function both in the short- (LVEF) and long-axis (MAPSE) deteriorated significantly following TAC in male mice. Females maintained the short-axis systolic function after TAC (Fig. 3A) but displayed a significant reduction of the long-axis function (Fig. 3C) compared with baseline values. The LV filling pressure, reflected by the ratio E/E′, increased after TAC in both sexes, although the rise was significantly higher in males than in females (Fig. 3E).

At baseline, the LVMI gravimetric or estimated by echocardiography was similar in intact male and female mice (Table 1, Fig. 1). Two weeks after TAC, the intact mice developed significant LV hypertrophy regardless of their sex (Table 1 and Fig. 2). In absolute terms, the gravimetric LVM was greater in TAC-males, but after indexation to body weight, this difference disappeared (Table 1).

### TAC-induced myocardial overexpression of remodeling-related genes was more pronounced in males than in females

**Figure 4:**
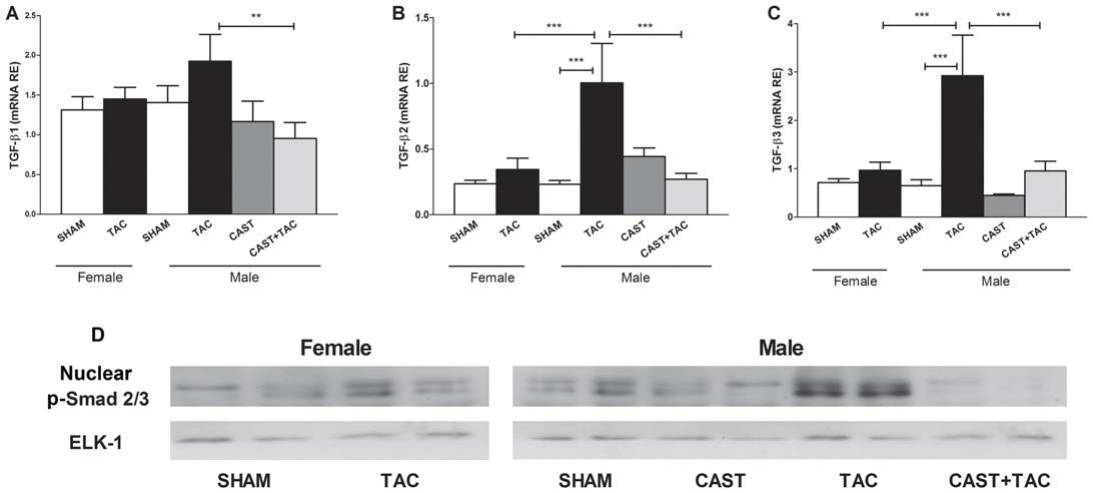
Effects of pressure overload on the myocardial expression of genes encoding TGF-βs and the nuclear protein accumulation of the canonical effectors, phospho-Smad2/3. mRNA relative expressions of TGF-β1 (**A**) , TGF-β2 (**B**) and TGF-β3 (**C**), and representative western blots showing the nuclear expression levels of phospho-Samd2/3 (loading control: ELK1) (**D**) in female and male mice under the following conditions: sham operated, castrated (CAST), subjected to aortic arch constriction (TAC), and castrated subjected to TAC (CAST+TAC). Data are expressed as the mean ± SEM. *p<0.05, **p<0.01, ***p<0.001 (ANOVA and Bonferroni post-hoc test).

The myocardial mRNA levels of TGF-β1, TGF-β2 and TGF-β3 (Fig. 4) as well as the nuclear protein levels of phosphorylated Smad2/3 increased significantly after TAC only in intact male mice (Fig. 4D). In females, neither the nuclear levels of phospho-Smad2/3 nor the TGF-β mRNA expression were significantly modified by the pressure overload condition.

**Figure 5:**
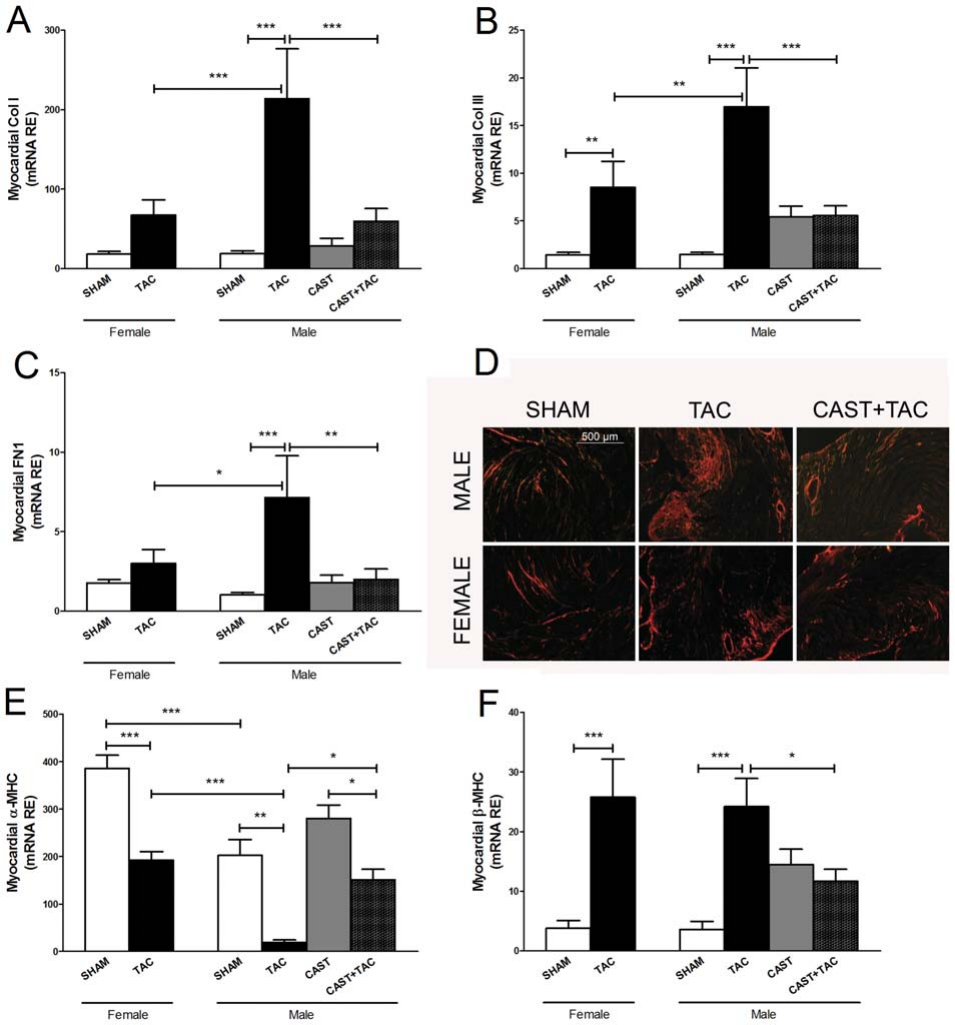
Effects of pressure overload on the myocardial expressions of extracellular matrix and sarcomeric remodeling-related elements. mRNA relative expressions of **A**: Collagen I (Col I); **B**: Collagen III (Col III); **C**: Fibronectin 1 (FN); **E**: α-Myosin heavy chain (α-MHC); **F**: β-Myosin heavy chain (β-MHC) in female and male mice under the following experimental conditions: Sham operated, castrated (CAST), subjected to aortic arch constriction (TAC), and castrated subjected to TAC (CAST+TAC). Data are expressed as the mean ± SEM. *p<0.05, **p<0.01, ***p<0.001 (ANOVA and Bonferroni post-hoc test). **D**: Representative histological sections illustrating myocardial tissue fibrosis (polarized light, picrosirius red stain, original magnification: 50×).

In parallel, extracellular matrix genes showed a significant up-regulation in TAC-males, reaching higher expression values than in TAC-females (Fig. 5). The changes observed in the mRNA expression of collagen were confirmed by picrosirius red histological staining (Fig. 5). The percentage of the area stained by picrosirius red was 2.3±0.7 fold higher in TAC-males than in TAC-females (p<0.001). The levels of α-MHC mRNA were significantly reduced only in TAC-males, whereas β-MHC mRNA was increased after TAC in both sexes to a similar extent (Fig. 5).

### TAC-induced myocardial fibrosis was prevented in males by systemic inhibition of TGF-β

**Figure 6:**
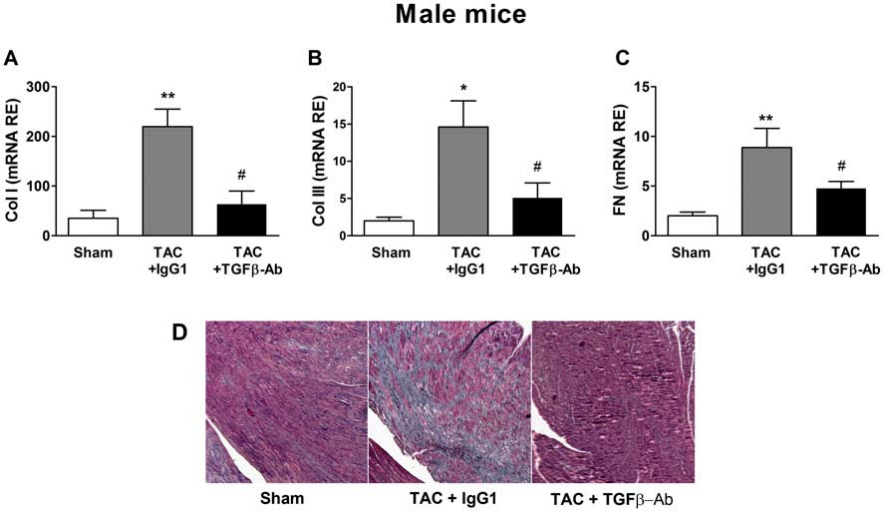
Effects of the treatment with a TGF-β neutralizing antibody on the extracellular matrix remodeling induced by pressure overload in male mice. Myocardial transcript levels of **A**: Collagen I (Col I); **B**: Collagen III (Col III) and **C**: Fibronectin 1 (FN); in male mice sham operated or subjected to aortic arch constriction (TAC) and treated either with IgG1 or with a neutralizing antibody that recognizes all the isoforms of TGF-β (TGFβ-Ab). Data are expressed as the mean ± SEM. *p<0.05, **p<0.01 versus Sham; #p<0.05 versus TAC+TGFβ-Ab (ANOVA and Bonferroni post-hoc test). **D**: Representative histological sections illustrating myocardial fibrosis in the interventricular septum region stained in blue by Massons'trichrome (50×).

A series of TAC-male mice were treated during the follow-up period with a neutralizing monoclonal antibody anti-TGF-β or with a control IgG1 immunoglobulin. TAC-males receiving TGF-β-Ab displayed markedly reduced fibrosis in comparison with TAC-males treated with IgG1, as indicated by Masson's trichrome staining of heart sections and the expression levels of genes encoding collagens and fibronectin (Fig. 6).

### Removal of circulating androgens by orchiectomy reduced the severity of TAC-induced myocardial remodeling in males

To assess the contribution of gonadal-released androgens to the less favorable cardiac remodeling observed in males compared with females, a series of male mice was subjected to orchiectomy two months before TAC or sham surgery. At baseline, orchiectomy affected heart size and function (Figs. 2 and 3). Compared with controls, CAST-males displayed the following characteristics: a) lower LV systolic and diastolic dimensions (Figs. 2D and F) with preserved LV geometry (Fig. 2H); b) higher basal LVEF (Fig. 3B); and c) lower LVMI (Table 1). Following TAC, castrated males displayed remodeling features identical to those of females, both functionally and geometrically. Male castration prevented the development after TAC of LV dilation (Fig. 2), long- and short-axis systolic dysfunction and LV filling pressure rise (Fig. 3). The LVM reached by CAST+TAC-males two weeks after TAC was lower (absolute or indexed LVM values) than in TAC-males (Fig. 2 and Table 1). However, as castrated males had lower LVMI than the controls at the time of TAC surgery, no differences between CAST+TAC- and TAC-male mice were evident in the relative increase of the postoperative LVMI.

In male mice, castration prevented TAC-induced overexpression of TGF-β isoforms as well as the nuclear accumulation of phospho-Smad2/3 proteins (Fig. 4). Accordingly, the transcripts levels of TGF-β targets such as collagen I, collagen III and fibronectin showed significantly lower expression levels in CAST+TAC-males than in TAC-males and similar values compared with females (Fig. 5). The changes observed in the mRNA expression levels of collagens were confirmed by picrosirius red histological staining (Fig. 5). The percentage of the area stained by picrosirius red was: TAC-males: 15.5±1.8% *vs* CAST+TAC-males: 6.3±1.2%; p<0.01.

To rule out whether myocardial remodeling in one-year-old females was influenced by residual ovarian estrogen production, a series of female mice was ovariectomized and subjected to TAC or sham surgery two months after castration. No significant differences between CAST-TAC-females and TAC-females were observed in any of the echocardiographic parameters analyzed or in the expression of remodeling-related genes (data not shown).

### Relationship between myocardial gene expression and LV function

To ascertain whether the changes in the myocardial expression of remodeling-related genes could provide mechanistic insights into the effects of pressure overload and castration on heart performance, we analyzed the existence of a relationship in male and female mice between gene expression and echocardiographic parameters of hypertrophy and function using linear regression analysis (Table 2). LV mass correlated negatively with α-MHC expression and directly with the expression of collagens I and III and fibronectin. LVEF correlated directly with α-MHC expression in all groups and was inversely related to the expression of collagens and fibronectin in males. MAPSE correlated in both sexes directly with α-MHC expression and inversely with the expression of fibrosis-related genes. The E/E′ ratio was directly related to the expression of collagens, fibronectin and β-MHC in male and female animals.

**Table 2:** Pearson's correlation coefficient values (R) obtained from the linear regression analyses correlating myocardial mRNA expression levels of collagen I (Col I), collagen III (Col III), fibronectin 1 (FN), β-Myosin heavy chain (β-MHC) and α-Myosin heavy chain (α-MHC) with the following echocardiographic and tissue Doppler parameters: LV mass; LV ejection fraction (LVEF); mitral annular plane systolic excursion (MAPSE); peak early transmitral flow velocity (E) to peak early myocardial tissue velocity (E′) ratio.

		*Col I*	*Col III*	*FN1*	*β-MHC*	*α-MHC*
**LV mass**	Female	0.40*	0.48**	0.43**	0.15	−0.60***
	Male	0.54***	0.54***	0.46***	0.13	−0.56**
**LVEF**	Female	−0.36	−0.24	−0.37	−0.05	0.45*
	Male	−0.69***	−0.65***	−0.64***	−0.39	0.41*
**MAPSE**	Female	−0.61***	−0.45***	−0.57***	−0.42	0.50**
	Male	−0.61***	−0.63***	−0.42*	−0.26	0.44*
**E/E′**	Female	0.69***	0.50*	0.52*	0.57*	0.11
	Male	0.69***	0.70***	0.53**	0.52*	−0.23

The correlations include individuals (minimum 25) from each experimental condition.

### Correlations in myocardial gene expression data

As shown in Table 3, the expression of the three TGF-β isoforms and their effectors Smad2 and Smad3 was correlated in both sexes directly and significantly with the expression of fibrosis-related genes and β-MHC; the expression of α-MHC displayed no significant relationship with that of TGF-βs (data not shown). Unexpectedly, β-MHC expression correlated significantly with that of collagen I (female: R = 0.52**; male: R = 0.59**), collagen III (female: R = 0.70***; male: R = 0.74***) and fibronectin (female: R = 0.54**; male: R = 0.76***).

**Table 3:** Pearson's correlation coefficient values (R) obtained from the linear regression analyses correlating myocardial mRNA relative expression levels of TGF-β1, TGF-β2 and TGF-β3 and their effector proteins Smad2 and Smad3 with the expression of collagen I (Col I), collagen III (Col III), fibronectin 1 (FN), and β-Myosin heavy chain (β-MHC).

		*Col I*	*Col III*	*FN*	*β-MHC*
***TGF-β1***	Female	R = 0.56***	R = 0.47**	R = 0.63***	R = 0.30
	Male	R = 0.56***	R = 0.51**	R = 0.54***	R = 0.42*
***TGF-β2***	Female	R = 0.60***	R = 0.56***	R = 0.58***	R = 0.43*
	Male	R = 0.80***	R = 0.69***	R = 0.55***	R = 0.60***
***TGF-β3***	Female	R = 0.54**	R = 0.70***	R = 0. 86***	R = 0.52**
	Male	R = 0.71**	R = 0.77***	R = 0.72***	R = 0.72***
***Smad2***	Female	R = 0.54**	R = 0. 52**	R = 0.74***	R = 0.64***
	Male	R = 0.39*	R = 0.40*	R = 0.51**	R = 0.63***
***Smad3***	Female	R = 0.58**	R = 0.52*	R = 0.38	R = 0.47*
	Male	R = 0.62**	R = 0.42*	R = 0.16	R = 0.46 *

The correlations include TAC-individuals (minimum 20) from each experimental condition.

### Exposure of cultured fibroblast NIH-3T3 and cardiomyocyte H9C2 cell lines to dihydrotestosterone leads to TGF-β mRNA overexpression

**Figure 7:**
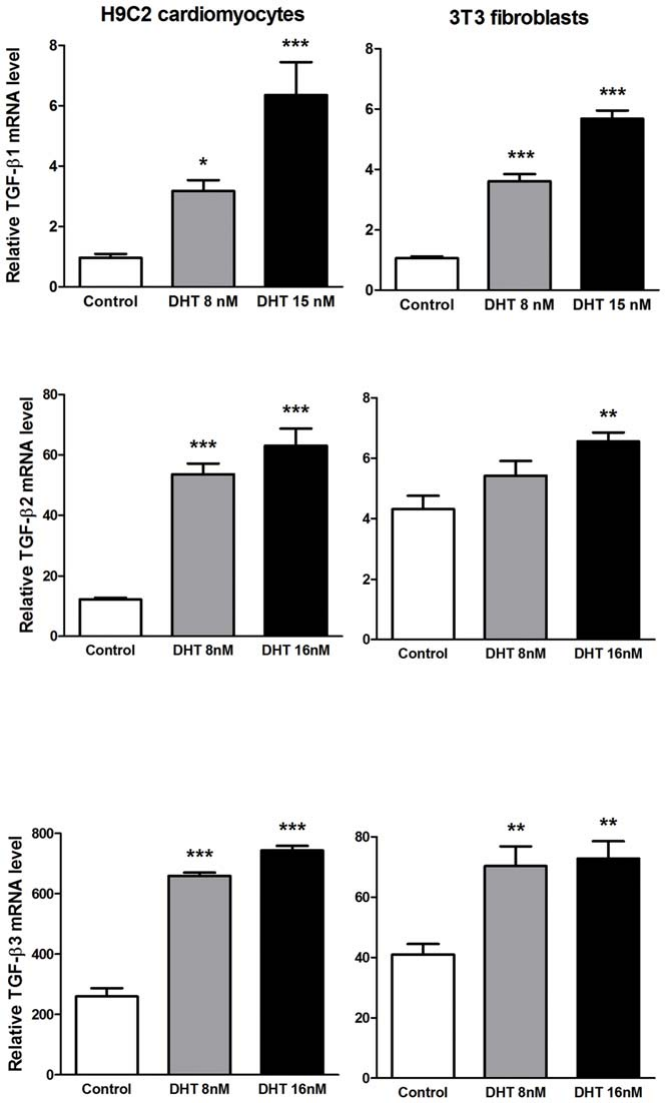
Effects of androgens on TGF-β mRNA expression in cultured H9C2 cardiomyocytes and NIH3T3 fibroblasts. Cells were exposed to dihydrotestosterone (DHT) for 16 h. Gene expression, determined by real-time PCR, is expressed as the fold induction. Data are the means ± S.E. of three experiments performed in duplicate. *p<0.05, ***p<0.001 vs. control cells (vehicle treated) (ANOVA and Bonferroni post-hoc test).

Exposure of either NIH-3T3 fibroblasts or H9C2 cardiomyocytes to physiological concentrations of DHT (8 and 15 nM) significantly increased the mRNA levels of TGF-β1, TGF-β2, and TGF-β3 (Fig. 7).

## Discussion

Sex-related differences in myocardial remodeling have been observed repeatedly in patients with LV pressure overload produced by AS [9]–[15]. However, although these clinical observations were obtained from older patients, experimental studies addressing sex differences in LV remodeling were typically performed in young animals [28], [29]. Our experimental design attempted to mimic more closely the clinical scenario by analyzing the differential remodeling characteristics of one-year-old male and female mice following pressure overload induced by TAC. Male mice at this age exhibited, like older men, circulating androgen levels that fell within the normal range for young adults. On the other hand, circulating estrogen levels in female mice at one year of age were reduced to values similar to those of males [30], as occurs in postmenopausal women.

Intact older male and female mice developed comparable levels of LV hypertrophy after TAC, which is similar to clinical observations where elderly patients with AS display equivalent LVMI irrespective of sex, provided that mass normalization is performed properly [9], [31]. Conversely, young female mice respond to pressure overload with a lower degree of hypertrophy than males, which was attributed to a protective effect of estrogens [30], [32]. Thus, in our study the absence of such a limiting hormonal mechanism in old females would equalize the hypertrophic response in both sexes. As observed in human studies, TAC-female mice developed hypertrophy with preserved LV chamber dimensions, whereas TAC-males showed a more dilated LV and a significantly greater reduction in systolic function in both the radial (reflected by LVEF) and longitudinal (reflected by MAPSE) axes. In females, LV long-axis function was moderately but significantly reduced after TAC, whereas the LVEF remained normal. The evaluation of longitudinal systolic and diastolic function indices has been the object of extensive interest in recent years, particularly in subjects with LV hypertrophy [33], [34]. There is a widespread consensus that longitudinal function deteriorates early in cardiac conditions such as pathological hypertrophy, even if short-axis function remains relatively preserved [35]–[37]. The long-axis function is the first to deteriorate under pressure overload because it is governed by the subendocardial layer of longitudinal muscle fibers, which is most vulnerable to increased systolic wall stress and usually the primary location of fibrosis onset [38]. In TAC-females, the LV filling pressure, as observed in its surrogate variable E/E′, rose significantly in the absence of LVEF deterioration. This phenomenon, frequently categorized in the clinical scenario as “pure” diastolic dysfunction, is more prevalent in women with AS than in men [39]. Nevertheless, often patients with “pure” diastolic dysfunction also have impaired longitudinal systolic function [40], as observed here in TAC-females, indicating the existence of mixed LV dysfunction.

Under our experimental conditions of age and pressure overload, orchiectomy prevented most of the remodeling responses induced by two-week TAC. Thus, hypertrophy reached lower values, the LV chamber dilation was lower, the radial and longitudinal systolic functions were less severely diminished, and the LV filling pressure did not increase. In summary, the original sex-related differences disappeared, and the echocardiographic behavior of castrated males did not differ significantly from that observed in females.

The sex-dependent echocardiographic differences of our animals were mirrored by the LV gene expression patterns of remodeling-related elements. Our results implicate TGF-βs in these differences, as all TGF-β isoforms and the nuclear expression of Smad2/3 were up-regulated in the LV from TAC-males but not TAC females. Moreover, treating TAC-males with a TGF-β neutralizing Ab inhibited the development of fibrosis.

Orchiectomy prevented LV up-regulation of TGF-βs along with collagens and fibronectin and it reduced the hemodynamic deterioration. Increased myocardial extracellular matrix deposition under transcriptional control by TGF-βs is a hallmark of the remodeling response to pressure overload [3]. Consequently, we observed strong positive relationships between transcript levels of all TGF-β isoforms and the mRNA levels of their target genes, i.e., collagens I and III and fibronectin. These data support that the circulating sex hormone background contributed to the male sex-related increase in fibrosis and subsequent LV dysfunction after TAC through a mechanism involving TGF-βs.

The androgen receptor (AR) is a ligand-dependent transcription factor that mediates androgen functions by regulating the expression of target genes. Multiple functional positive and negative androgen-response elements (AREs) are present in the promoter of the TGF-*β*1 gene [41]. Androgens can directly regulate TGF-β1 expression through these AREs positively or negatively depending on cell type or context. Several reports show that androgens induce the production of TGF-β1 in a number of non-cardiac cell types [42]–[44]. Here, cultured fibroblast and cardiomyocyte cell lines responded to physiological concentrations of DHT with TGF-β mRNA overexpression. In mice, the myocardial expression levels of TGF-β correlated directly with the AR mRNA levels (data not shown). Based on these findings, we suggest that circulating androgens could contribute to the transcriptional activation of genes encoding TGF-βs in the pressure overloaded myocardium and that TGF-βs might in turn mediate several effects of androgens, including pathological fibrosis.

Clinical studies in patients with LV hypertrophy and AS have found that MAPSE, which is a powerful predictor of outcome, correlated tightly with myocardial fibrosis, as assessed by magnetic resonance imaging and histology [36]. In our pressure overload model, MAPSE is closely related to the expression of fibrosis-related genes. The physiological meaning of this relationship is supported by the significant correlation between extracellular matrix gene expression and E/E′ that is a current index of diastolic pressure closely related with fibrosis [45], [46] . In line with the more severe hemodynamic dysfunction, collagens and fibronectin were more strongly up-regulated in males than in females. Furthermore, orchiectomy significantly prevented the increase in LV filling pressure and its structural fibrotic substrate.

As typically occurs in small animals [47], TAC-mice reacted to the pressure overload by re-expressing the fetal β-MHC isogene and reducing the expression of the adult predominant isoform, i.e., α-MHC. The transcriptional repression of α-MHC appeared unrelated to TGF-β signaling, whereas the transcription of β-MHC seemed to be under the control of this pathway, as previously reported in AS patients [9], [10]. The expression of α-MHC but not of β-MHC correlated with the severity of hypertrophy and the decline in systolic performance. In contrast, the expression levels of β-MHC displayed a strong correlation with fibrosis-related genes and with the degree of diastolic dysfunction. These results are in agreement with the notion of Pandya et al. [48] that β-MHC in the mouse heart is a marker of fibrosis rather than hypertrophy. In these author's experiments, β-MHC over-expression during cardiac hypertrophy and normal aging occurs in subsets of myocytes clustered within areas of fibrosis rather than in all hypertrophic cells. In our study, castration consistently prevented TAC-induced re-expression of β-MHC and overexpression of fibrosis-related genes without abolishing the increase in LV mass.

The effect of androgens on the human cardiovascular system is a matter of debate. Although androgens are responsible for several beneficial effects on the cardiovascular system [49], increasing evidence suggests that they can also exert detrimental effects [20]. Anabolic androgenic steroids have consistently been shown to impair LV diastolic and systolic functions [18], [19] in humans, and these clinical studies are supported by pathological evidence of increased myocardial collagen content after exposure to these drugs [17]. Similarly, rats subjected to exercise training and treated with anabolic steroids develop a maladaptive remodeling with deterioration of cardiac performance [21]. In a rat model of myocardial infarction, exogenous testosterone exerts deleterious effects on LV remodeling, contributing to a deterioration of systolic function and an increase in the cardiac rupture rate [22].

Recent reports show that physiological gonadal androgens play a critical role in the cardiomyopathy displayed by males, but not females, of several strains of genetically modified rodents. Thus, transgenic mice overexpressing β2-adrenergic receptor [23], mice lacking the gene encoding guanylyl cyclase-A [24] and heterozygous Ren-2 rats [25] develop spontaneously during adulthood a pathological cardiac phenotype characterized by hypertrophy and fibrosis, which associates progressive LV dilatation and systolic dysfunction [23]. In all these models as in ours, the pathological cardiac phenotypes were associated with up-regulation of TGF-β, and both features were prevented by orchiectomy.

In contrast with the above findings, male mice lacking androgen receptors (ARKO mice) respond to a hypertrophic stimulus (angiotensin II administration) with less hypertrophy but more severe dysfunction and prominent fibrosis in association with a higher activation of the TGF-β/Smad2 pathway compared with wild-type animals [50]. However, the complex phenotype (testicular feminization mutation syndrome) of global ARKO male mice makes it difficult to differentiate the direct phenotypic consequences of the lack of nuclear AR in myocardial cells from indirect off-target effects. The need for further studies to clarify the role of AR in myocardial remodeling appears obvious, and the cell-specific conditional knockout approach would be particularly useful for such a purpose.

In summary, our results show that one-year-old male mice develop a less favorable myocardial remodeling (in terms of fibrosis, geometry and hemodynamic function) than females in response to pressure overload, which is associated with higher myocardial expression levels of TGF-βs. Orchiectomy prevented TGF-β up-regulation, fibrosis and dysfunction, supporting that TGF-βs can act downstream of androgens to promote fibrosis. We suggest that in older individuals, the detrimental effects of circulating androgens in males, rather than the protective actions of estrogens in females, contribute to sex-related myocardial remodeling differences.

## Supporting Information

Figure S1 **Representative echocardiographic recordings.** **A**: Parasternal short-axis M-mode tracings of the left ventricle at the midpapillary level. LVESd: Left ventricular end-systolic dimension; LVEDd: Left ventricular end-diastolic dimension; PWT: posterior wall thickness; IVST: interventricular septum thickness. Four-chamber M-mode tracings showing the mitral annular plane systolic excursion (MAPSE). **C**: Peak early transmitral flow velocity (E) from pulsed Doppler tracing. **D**: Peak early myocardial tissue velocity (E′) of tissue Doppler tracing from the posterior wall in the parasternal short-axis view. **E**: 2D-guided pulsed Doppler recording of the coarctation gradient in a TAC mouse at the aortic arch, immediately distal to the constriction.
(DOC)
