# Supplementary material for: Androgens Contribute to Sex Differences in Myocardial Remodeling under Pressure Overload by a Mechanism Involving TGF-β
Source: PLoS One. 2012 Apr 25;7(4):e35635. doi: 10.1371/journal.pone.0035635 (PMC3338422; doi:10.1371/journal.pone.0035635)

**Supplementary figure S1**

Representative echocardiographic recordings. **A**: Parasternal short-axis M-mode tracings of the left ventricle at the midpapillary level. LVESd: Left ventricular end-systolic dimension; LVEDd: Left ventricular end-diastolic dimension; PWT: posterior wall thickness; IVST: interventricular septum thickness. Four-chamber M-mode tracings showing the septal mitral annular plane systolic excursion (MAPSE). **C**: Peak early transmitral flow velocity (E) from pulsed Doppler tracing. **D**: Peak early myocardial tissue velocity (E') of tissue Doppler tracing from the posterior wall in the parasternal short-axis view. **E**: 2D-guided pulsed Doppler recording of the coarctation gradient in a TAC mouse at the aortic arch, immediately distal to the constriction.


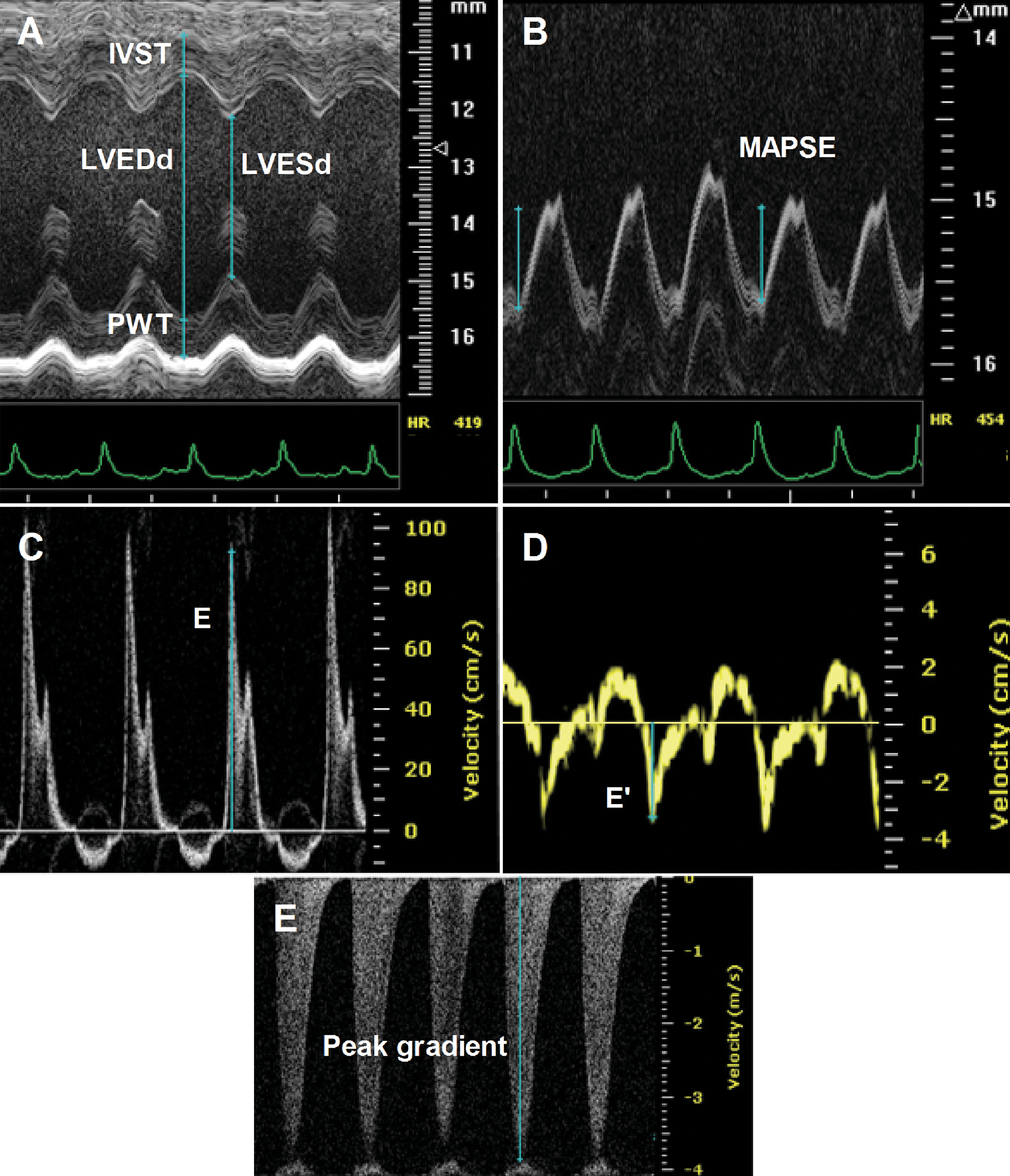

Supplement: Figure S1 — Representative echocardiographic recordings. A: Parasternal short-axis M-mode tracings of the left ventricle at the midpapillary level. LVESd: Left ventricular end-systolic dimension; LVEDd: Left ventricular end-diastolic dimension; PWT: posterior wall thickness; IVST: interventricular septum thickness. Four-chamber M-mode tracings showing the mitral annular plane systolic excursion (MAPSE). C: Peak early transmitral flow velocity (E) from pulsed Doppler tracing. D: Peak early myocardial tissue velocity (E′) of tissue Doppler tracing from the posterior wall in the parasternal short-axis view. E: 2D-guided pulsed Doppler recording of the coarctation gradient in a TAC mouse at the aortic arch, immediately distal to the constriction. (DOC) [file pone.0035635.s001.doc]
